# Psychosocial impact of COVID-19 on caregivers and adolescents and young adult survivors of childhood cancer

**DOI:** 10.1002/pbc.30291

**Published:** 2023-03-23

**Authors:** Sara King-Dowling, Shannon N. Hammer, Haley Faust, Rebecca Madden, Sarah Drake, Annisa Ahmed, May Albee, Janet A. Deatrick, Lauren Daniel, Ahna Pai, David Freyer, Alexandra M. Psihogios, Lamia P. Barakat, Lisa A. Schwartz

**Affiliations:** 1 Division of Oncology, Children’s Hospital of Philadelphia, Philadelphia, Pennsylvania, USA; 2 Patient & Family Wellness Center in the Cancer and Blood Diseases Institute, Division of Behavioral Medicine & Clinical Psychology, Cincinniati Children’s Hospital Medical Center, Cincinnati, Ohio, USA; 3 Department of Family and Community Health, University of Pennsylvania School of Nursing, Philadelphia, Pennsylvania, USA; 4 Department of Psychology, Rutgers University-Camden, Camden, New Jersey, USA; 5 Department of Pediatrics, University of Cincinnati College of Medicine, Cincinnati, Ohio, USA; 6 Cancer and Blood Disease Institute, Children’s Hospital Los Angeles, Los Angeles, California, USA; 7 USC Norris Comprehensive Cancer Center, Los Angeles, California, USA; 8 Department of Medical Social Sciences, Feinberg School of Medicine, Northwestern University, Chicago, Illinois, USA; 9 Department of Pediatrics, University of Pennsylvania Perelman School of Medicine, Philadelphia, Pennsylvania, USA

**Keywords:** adolescent, cancer, caregivers, COVID-19, psycho-oncology, young adult

## Abstract

**Background:**

Caregivers and adolescents and young adult (AYA) cancer survivors may be at greater psychosocial risk from the COVID-19 pandemic than healthy peers due to complex and traumatic medical histories. This study describes COVID-19-related event exposures, impact, and distress among a large sample of caregivers and AYA cancer survivors and the relationship of these variables to demographic and cancer characteristics.

**Procedure:**

From May 2020 to December 2021, 422 caregivers and 531 AYA survivors completed the COVID-19 Exposures and Family Impact Survey (CEFIS) and CEFIS-AYA, respectively. Total COVID-19-related exposures, average COVID-19-related impact, and COVID-19-related distress were calculated. Conventional content analysis was used to analyze free-text responses about the negative and positive effects of COVID-19.

**Results:**

Caregivers and AYA reported an average of 7.4–7.8 COVID-19 exposures to pandemic-related events and a slightly negative impact of COVID-19 across psychosocial domains, with some positive impacts reported. COVID-19-related distress was moderate and clinically meaningful (4.9–5.2/10) for AYA and caregivers. Racial and ethnically minoritized AYA and caregivers reported higher COVID-19-related distress than non-Hispanic white caregivers. For AYA, distress was also higher among female, college-age (18–22 years), and long-term survivors compared with males, younger AYA, White and those recently off treatment. CEFIS outcomes remained relatively stable over time.

**Conclusions:**

COVID-19 had a significant and consistent negative impact on caregivers and AYA survivors. Racial and ethnically minoritized families and female, college-age, and long-term AYA survivors may require additional psychosocial support. Assessing for COVID-19 impact and distress is important in pediatric oncology to evaluate adjustment and plan targeted interventions.

## 1 | INTRODUCTION

COVID-19 has impacted the well-being of individuals, especially children, adolescents and young adults (AYA), and their caregivers.^1–4^ For cancer survivors under the age of 30 years and their caregivers, exposures to potentially traumatic events throughout the COVID-19 pandemic (e.g., isolation, medical risk/illness) may have been compounded by existing cancer-related experiences, challenges, and medical vulnerabilities. As the pandemic and related restrictions have eased, it is now critical to examine traumatic aspects of COVID-19 and assess the persistent impact of pandemic-related events on caregivers and AYA using a trauma-informed lens^5^ to enhance understanding of the psychosocial effects beyond the first year of the pandemic, inform optimal support and trauma-informed care, and consider implications related to possible future pandemics.

A trauma-informed lens can account for the cumulative traumas and stressors faced by survivors living with and beyond cancer during the COVID-19 pandemic. For example, they may be at increased risk for a more severe course of COVID-19 due to the acute and long-term side effects of their intensive cancer treatment.^6^ As such, various guidelines^7–9^ advise cancer survivors to take extra precautions to avoid COVID-19. Psychosocial and cognitive late effects of cancer may be exacerbated due to COVID-related worries about contracting/transmitting the virus,^10,11^ disruptions in cancer care and support services,^12,13^ distance education challenges,^14,15^ social isolation,^3^ and increased financial strain caused by the pandemic.^16^ Emerging research describes elevated anxiety, greater distress, and poorer emotional functioning in caregivers^11,12,15,17^ and AYA survivors^13,15,18–20^ due to COVID-19. However, this evidence has primarily relied on non-validated, atheoretical, and/or non-COVID-specific measures of psychosocial functioning early in the pandemic.^10–12,14–21^ Furthermore, we have a limited understanding of the characteristics of caregivers and AYA impacted by cancer who may be at the greatest risk of COVID-related negative outcomes.

The COVID-19 pandemic has also amplified health disparities among both healthy populations and cancer survivors, another potentially traumatic trigger for those affected. In the United States, there have been higher rates of COVID-19 infection, hospitalization, and death among racially and ethnically minoritized groups both with and without cancer and more disruptions to cancer-related care compared with white individuals.^22–26^ The pandemic has also highlighted gender inequities, with female caregivers^27^ and AYA,^2,28^ as well as gender-diverse young adults,^29,30^ reporting higher rates of mental health challenges during COVID-19 compared with males. There is limited research examining if these psychosocial disparities are also present among caregivers and AYA cancer survivors during COVID-19.

To understand the impact of COVID-19 in pediatric and AYA oncology through a trauma-informed lens, we applied our validated measure of COVID-19 exposures and impact (CEFIS: COVID-19 Exposures and Family Impact Survey)^31,32^ to a diverse sample, including caregivers and AYA cancer survivors. This was a result of a broader effort at our Cancer Center to add the CEFIS to ongoing studies to assess COVID-19-related exposures and psychosocial impact to understand the impact of COVID-19 on research and patient/caregiver well-being and guide trauma-informed care. The aims of this cross-sectional study were to (1) describe self-reported exposures to events related to the COVID-19 pandemic and the psychosocial impact of COVID-19 on caregivers and AYA cancer survivors across the first 20 months of the COVID-19 pandemic and (2) determine if these outcomes differ by demographic characteristics or cancer history.

## 2 | METHODS

### 2.1 | Participants

This study compiled cross-sectional data across seven ongoing research studies and one clinical program led by Behavioral Oncology at the Children’s Hospital of Philadelphia from May 2020 to December 2021. These studies recruited caregivers and/or AYA survivors (age 15–29 years) across a variety of cancer diagnoses and treatment statuses, including active treatment, recently off treatment (<2 years), and long-term survivorship (≥2 years off treatment). One ongoing long-term survivorship study is multisite and included AYA survivors and their caregivers recruited from Cincinnati Children’s Hospital Medical Center and Children’s Hospital Los Angeles. Each study received IRB approval to add the CEFIS to the existing research protocols. A full breakdown of each study purpose, eligibility criteria and recruitment methods can be found in Table S1. In general, caregivers were eligible to participate if they had a child (<30 years) with a confirmed cancer diagnosis (either on or off treatment) and were English or Spanish speaking. AYA were eligible if they had a confirmed cancer diagnosis (on or off treatment), were between the ages of 15–29 years, English speaking and cognitively able to complete the CEFIS-AYA independently. For all studies, consent was obtained from caregivers and AYA ≥ 18 years old (assent for AYA under the age of 18 years), for Spanish-speaking caregivers, consent was obtained via interpreter. The clinical program received IRB exemption to include clinically collected information in research.

### 2.2 | Measures

#### 2.2.1 | Demographics and cancer characteristics

Caregiver and AYA demographics (age, sex, race, ethnicity, income) were collected via self-report and cancer diagnosis and treatment status were obtained via the electronic health record.

#### 2.2.2 | COVID-19 Exposures and Family Impact Survey

Caregivers and AYA completed the CEFIS^31^ or CEFIS-AYA,^32^respectively. The CEFIS and CEFIS-AYA are available in English and Spanish and have been validated in a large sample of caregivers and AYA across the United States^31–33^ from a variety of patient populations, including a subset of the current oncology sample, and include domains consistent with a medical traumatic stress framework. Both versions contain 3 parts. *Part 1: Exposure* consists of 25–28 items with yes/no responses that measure potentially traumatic “exposures” to the COVID-19 virus and pandemic-related events (e.g., stay-at-home orders, school closures, loss of income, hospitalization of family members, etc.). The total *exposure* score (Part 1) is calculated as the total number of “yes” responses, with higher scores indicating greater exposures (CEFIS range 0–25, CEFIS-AYA range 0–28). *Part 2: Impact* assesses the impact of COVID-19 on family, emotional, and physical well-being. The majority of items use a 4-point Likert-type scale from 1 (made it a lot better) to 4 (made it a lot worse) and a “Not Applicable” (N/A) option. Mean impact (Part 2) is calculated as the average impact rating (excluding the distress items, CEFIS: 10 items, CEFIS-AYA: 15 items). Calculation of a valid mean impact score required a minimum 7 and 11 valid responses for caregivers and AYA, respectively.^31,32^ A mean impact score > 2.5 indicates a negative valence and < 2.5 a positive valence. *Part 2: COVID-19-related distress* is examined and scored separately^31–33^ and measured on a 10-point scale that includes 1 item asking about distress for the CEFIS-AYA and two items on the CEFIS representing caregiver distress and proxy report of child distress. Higher scores on each scale indicate greater exposure, more negative impact, and more distress. *Part 3* consists of one open-ended question: “Please tell us about other effects of COVID-19 on you and your family, both negative and/or positive.” Exposures and impact subdomain scores were also calculated.

### 2.3 | Statistical analysis

CEFIS scores were examined via descriptive statistics. Caregivers and AYA who did not meet the minimum valid responses for the impact scale were excluded from the impact analyses. Comparisons between groups were made via independent samples *t*-tests and one-way ANOVAs followed by Bonferroni post-hoc tests where indicated. Effect sizes (Cohen’s *d* or eta-squared) were calculated. Caregivers and AYA who identified as Hispanic and/or Black, Asian, another race or multiracial were classified as Black, Indigenous and People of Color (BIPOC) for analysis. Due to the small number of AYA (*n* = 5) and caregivers (*n* = 1) identifying as another gender identity, these individuals were not included in the gender analysis and summarized descriptively.^34^ Associations between household income, caregiver age, exposures, impact, and distress as well as associations between CEFIS scores and time since the declaration of the pandemic (March 11, 2020) were examined via Pearson’s *r* correlations. Exploratory analysis examined which exposure and impact subdomains were most strongly associated with distress.

Five *Part 1* questions assess direct experience with the COVID-19 virus (e.g., direct exposure, hospitalization, etc.) and, when endorsed, allow respondents to specify who in the family was exposed. Responses were coded and summarized descriptively. Using conventional content analyses, free-text responses from *Part 3* were coded into categories and subcategories by four independent raters (SKD, SH, HF, and RM) and discussed with a qualitative expert (JD) to obtain a final consensus.^35^ Spanish free-text responses were translated into English by a native Spanish speaker before coding and subsequently verified by a second translator.

## 3 | RESULTS

### 3.1 | Participant characteristics

Four hundred and twenty-two caregivers and 531 AYA completed the CEFIS and CEFIS-AYA, respectively (see Table 1 for sample characteristics). Twelve pairs of caregivers were from the same family and approximately half of the AYA (*n* = 286, 53.9%) had a matching caregiver included. Approximately one-third of the caregivers (*n* = 136) had a younger child (age <15) or AYA who did not complete the CEFIS-AYA.

### 3.2 | Exposures to COVID-19-related events

**FIGURE 1:**
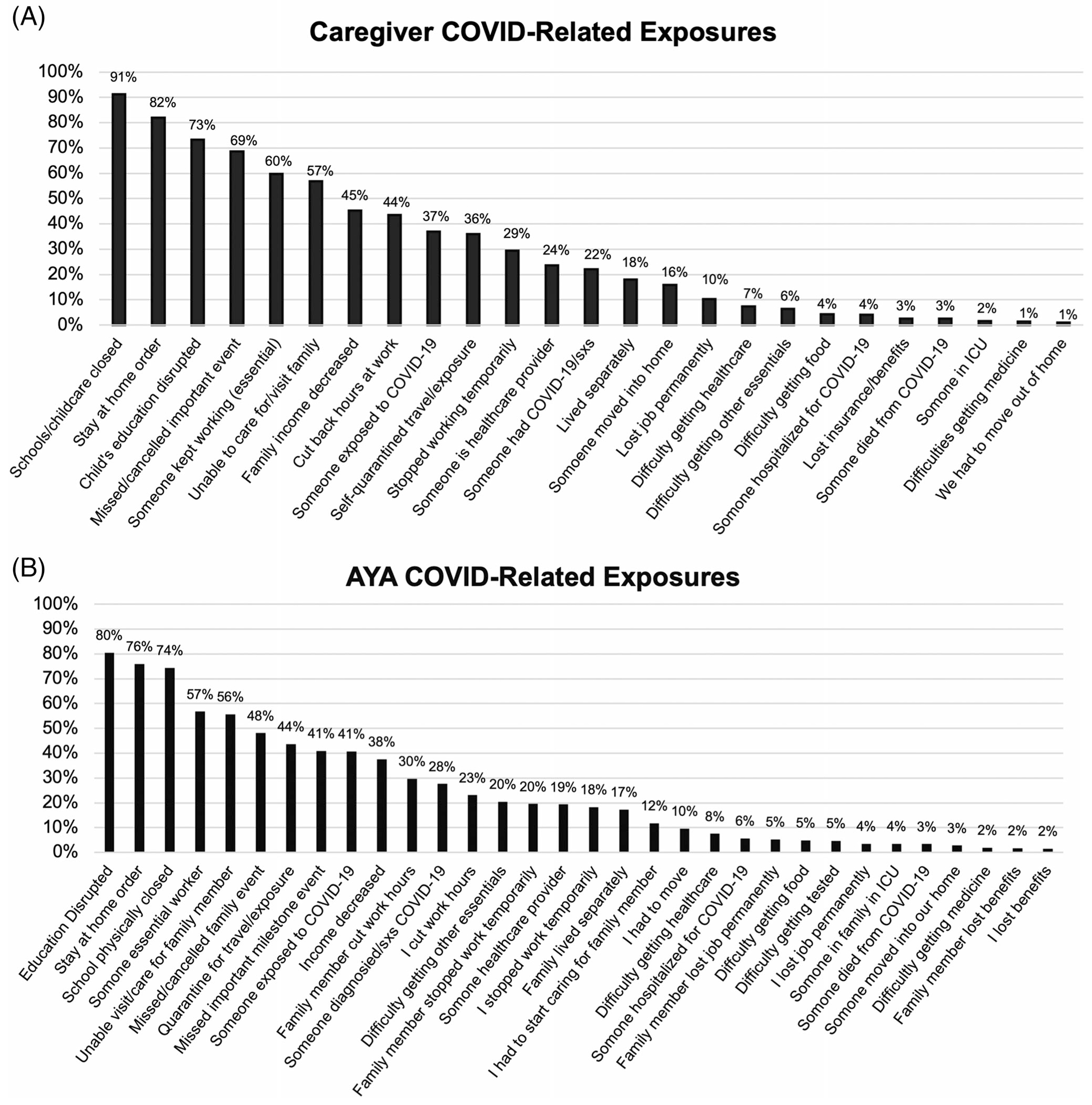
Caregiver (A) and AYA (B) COVID-19-related exposures. ICU, intensive care unit.

Caregivers and AYA reported an average of 7.4 (SD = 3.0, range 0–18) and 7.8 (SD = 3.3, range 0–20) exposures to COVID-related events, respectively (Figures 1A and B). Most frequent exposures (>70%) were school-related disruptions/closures and stay-at-home orders. Many reported direct exposure to the COVID-19 virus within their families, especially among caregivers/AYA’s parents, with more severe infections reported among caregivers’ parents (AYA’s grandparents) or other extended relatives (Figures S1A and S1B). No children and only one AYA were reported to be hospitalized due to COVID-19.

### 3.3 | COVID-19 impact

**FIGURE 2:**
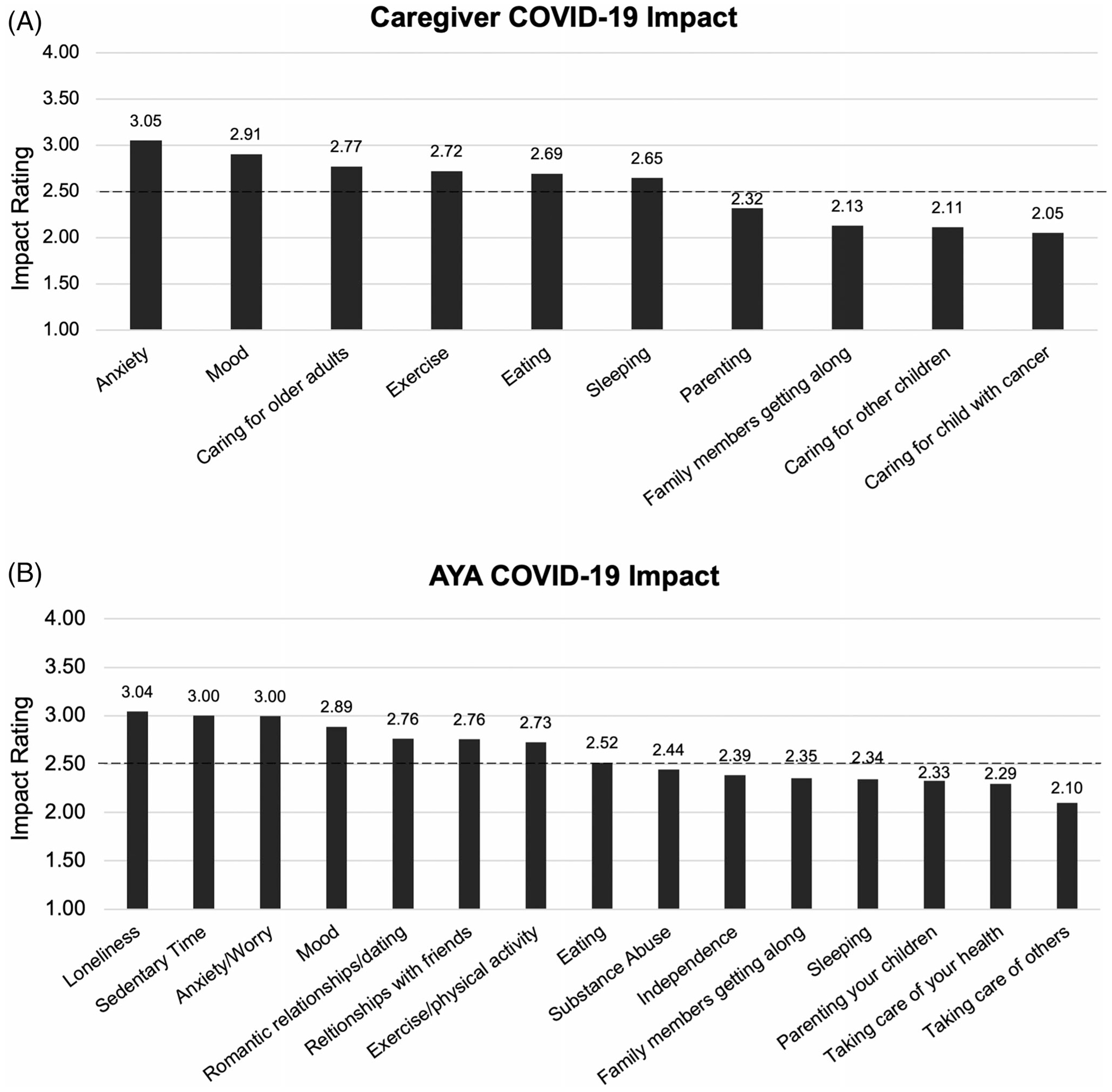
Caregiver (A) and AYA (B) COVID-19 impact ratings. Note: A mean impact score > 2.5 indicates a negative impact and < 2.5 a positive impact.

Valid mean impact scores were calculated for caregivers (*n* = 301, 87.5%) and AYA (*n* = 398, 74.6%). Caregivers and AYA reported an overall slightly negative impact of COVID-19 (caregivers: *M* = 2.53; SD = 0.63; AYA: *M* = 2.63; SD = 0.58). Items pertaining to emotional and social well-being were most negatively impacted whereas family relationships and caregiving were more positively impacted by COVID-19 (Figures 2A and B).

### 3.4 | COVID-19-related distress

Moderate levels of COVID-related distress were reported across all groups (caregivers: *M* = 5.19, *SD* = 2.32, caregiver’s child(ren) (proxy): *M* = 5.18, SD = 2.37; AYA: *M* = 4.89, SD = 2.39).

### 3.5 | Demographic associates of COVID outcomes

#### 3.5.1 | Gender

Female caregivers and AYA reported greater impact and female AYA also reported more exposures compared with males (see Table 2). Caregiver distress did not differ by gender; however, AYA distress and caregiver’s proxy reports of their child’s distress were higher for female respondants compared with males. Although not able to be included in the statistical analyses, AYA identifying as *other* gender identity (*n* = 5) reported high exposures *M* = 9.20 (SD = 4.66), mean impact *M* = 3.43 (SD = 0.41), and distress *M* = 6.20 (SD = 2.17).

#### 3.5.2 | Age

Caregiver age was not associated with COVID-19 exposures, impact, or distress (rs < .1, ps > .05); however, a significant age effect emerged for AYA. College-age AYA (age 18–22 years) compared with the adolescent age group (15–17 years) had greater COVID-related exposures (*M* = 8.10 vs. 7.12, *F* = 4.47, *p* = .012, *η*^2^ = .010) and distress (*M* = 5.20 vs. 4.40, *F* = 6.63, *p* = .001, *η*^2^ = .025) but there were no significant differences for older AYA (age 23–28 years).

#### 3.5.3 | Income

For caregivers that reported household income (62.6%), income was not associated with impact or distress but was negatively associated with COVID-related exposures (*r* = −.16, *p* = .006).

#### 3.5.4 | Race and ethnicity

Non-Hispanic white (NHW) caregivers and AYA reported lower levels of distress compared with individuals identifying as BIPOC. Conversely, mean impact ratings were higher (more negative valence) for NHW caregivers compared wth BIPOC caregivers (see Table 3).

### 3.6 | Cancer history associates of COVID outcomes

#### 3.6.1 | Cancer type

For caregivers, those who had children with central nervous system (CNS) tumors reported greater COVID-19 impact (*M* = 2.88, SD = 0.42) compared with leukemia/lymphoma (*M* = 2.43, SD = 0.63) or solid tumors (*M* = 2.58, SD = 0.65, *F* = 7.88, *p* < .001, *η*^2^ = .050) (see Table S2). A similar nonsignificant trend for higher COVID-19 exposures (*F* = 2.60, *p* = .076, *η*^2^ = .012) and children’s distress (*F* = 2.61, p = .075, *η*^2^ = .012) for caregivers of children with CNS tumors was also found. For AYA, no significant differences in COVID-19 outcomes by cancer type emerged (*p*s > .05).

#### 3.6.2 | Treatment status

For caregivers, the treatment status of their children was not associated with CEFIS outcomes. For AYA, a significant main effect for treatment status was found (*F* = 8.14, *p* < .001, *η*^2^ = .030), with long-term survivors (>2 years off treatment) reporting greater COVID-related distress (*M* = 5.03, SD = 2.34) compared with AYA recently off treatment (*M* = 3.15, SD = 2.11, *p* = .002), but not significantly different than AYA on treatment (*M* = 4.11. SD = 2.62) (see Table S3). A similar nonsignificant trend emerged for COVID-19 exposures with long-term AYA survivors also reporting the greatest exposures (*F* = 2.58, *p* = .076, eta-squared = .010).

### 3.7 | Relationships among COVID-19-related exposures, impact, and distress

Significant correlations were observed among the CEFIS domains (*r*s .3 to .4, *p*s < .001). When exploring which exposure and impact subdomains were most associated with distress, all but essential worker designation were significant correlates, with the strongest associations between access to essentials (caregivers: *r* = .219, *p* < .001, AYA *r* = .237, *p* < .001) and personal well-being (caregivers *r* = .410, *p* < .001; AYA emotional well-being *r* = .405, *p* < .001, AYA physical well-being *r* = .359, *p* < .001).

### 3.8 | COVID-19-related exposures, impact, and distress over time

Over the study period (May 2020–Dec 2021), reporting of COVID-related impact and distress remained relatively stable across participants (Figures S2A and S2B), with no significant association with time. Only caregiver-reported exposures to COVID-19-related events increased significantly since the start of the pandemic (*r* = .121, *p* = .013).

### 3.9 | Qualitative results

Overall, 322 caregivers (76.3%) and 433 AYA (81.5%) provided an open-ended text response describing any other negative and/or positive effects of COVID-19 for themselves, their child/ren, and their families. Of these, 29 caregivers (9.0%) and 66 AYA (15.2%) indicated “none/NA” and were not further coded. The remaining caregivers and AYA reported effects of COVID-19 across 9 main categories including family, lifestyle, education, social well-being, emotional well-being, work, health behaviors, physical health, and developmental. See Table 4 for the frequency of main categories and notable quotes across caregivers and AYA.

Most of the identified categories and subcategories complement and illustrate the quantitative findings. For example, the most common caregiver-reported effects of COVID-19 were related to education (school arrangements; learning) and family (time; relationships; household management), while the most common AYA-reported effects were related to family (time; relationships) and lifestyle (regular/leisure activities; milestones/major life events; living arrangements). Qualitative analysis identified additional effects of COVID-19 that were not captured in *Parts 1* and *2* of the CEFIS, such as the behavioral response to the pandemic (e.g., mask wearing) and perceived risk of infection or uncertainty about the virus. See Table S1 for definitions and frequencies of categories and subcategories and additional notable quotes.

## 4 | DISCUSSION

This is one of the largest pediatric and AYA oncology studies to date characterizing COVID-19-related exposures, impact, and distress using a psychometrically validated, COVID-specific survey. Additional strengths include the assessment of a medically and demographically diverse sample, inclusion of both caregiver and AYA report, and collection of quantitative and qualitative data that reflect cumulative trauma exposures and impact. On average, caregivers and AYA reported seven to eight exposures to COVID-19 and related events, an overall negative impact of COVID-19 and clinically meaningful levels of COVID-related distress, with some positive impacts highlighted. Results support a trauma-informed framework as greater COVID-19-related exposures were associated with poorer psychosocial outcomes. School/education disruptions and stay-at-home orders were the most frequently experienced exposures and psychosocial impacts were most negative for anxiety, loneliness, sedentary behavior, and mood, and most positive for caregiving-related activities. Qualitative responses provided context and support for the quantitative findings and was consistent with other qualitative research among AYA.^36^ Despite small increases in COVID-19-related event exposures for caregivers across time, the psychosocial impact of COVID-19 on caregivers and AYA survivors remained relatively stable from May 2020 to December 2021, suggesting consistent impact and distress throughout various stages of the pandemic.

Our results supported significant demographic differences among COVID-19 exposures, impact and distress. Reports of COVID-19-related distress were higher among caregivers and AYA from racial and ethnic minoritized groups, further highlighting disparities in the psychosocial impact of this pandemic.^37^ That caregivers from racially and ethnically minoritized groups reported less negative impact of COVID-19, despite higher levels of COVID-related distress, may be a factor of higher levels of baseline distress extending beyond the pandemic. Female caregivers and AYA experienced a greater negative COVID-19 impact, with female AYA also reporting more exposures and distress compared with male AYA. This may be due to a variety of factors including greater caregiving responsibilities, greater losses in income, and increased social media use among females.^27,38^ These differences by AYA gender align with the CEFIS-AYA validation,^32^ yet differ from the CEFIS caregiver validation in which male caregivers reported greater exposures and no gender differences in impact. It is possible that over this longer monitoring period, the impact has become more negative for female caregivers and exposures have evened out.^27,39^ Consistent with prior findings,^32^ college-age adults (aged 18–22 years) had the greatest exposures and highest distress levels and may be at the greatest risk of poor psychosocial outcomes compared with younger and older AYA. This may reflect the significant disruptions to daily life reported in college-age samples, including abrupt campus evacuations and mandated relocation for students, interruptions to education, substantial academic frustrations, and greater fears of contracting COVID-19 from their peers.^36,40,41^

This large oncology sample allowed further exploration of differential exposures and psychosocial impact based on cancer type and treatment status. Qualitative data highlighted the combined impact of cancer and COVID-19 with specific fears, uncertainty, and distress due to current or past cancer history, but also a sense of shared experience and understanding for those who already had to isolate and/or miss school. COVID-19 had the largest negative impact on caregivers of CNS tumor survivors, although this pattern was not observed among AYA. This partially supports prior research^42^ that found older adult survivors of childhood cancer with CNS involvement were at greater risk of social isolation and unemployment due to COVID-19 compared with survivors of non-CNS cancers. Survivors of childhood brain tumors are at greatest risk for neurocognitive late effects that warrant a continuation of caregiving and support services into adolescence and adulthood.^43^ As disruptions in child(ren)’s education was one of the most universal and frequently reported exposures, this may have placed extra burden on those caring for survivors of CNS tumors who are more likely to require academic supports. Regarding treatment status, no associations were found for the caregiver sample, but long-term AYA survivors reported higher distress than AYA more recently off treatment.^10^ This may be due to COVID-19 eliciting symptoms of posttraumatic stress in long-term survivors due to reminders of social isolation and medical vulnerability, although these findings should be interpreted with caution due to the relatively small sample of on-treatment and recently off-treatment AYA in the sample. Future work examining other cancer-related factors, including late effects, treatment length and intensity as well as perceived COVID-19 risk will be important to further understand how cancer experiences may influence reactions to COVID-19.

By pooling cross-sectional data across an extended time frame, we were able to explore time effects which suggest sustained impact and distress of the COVID-19 pandemic. Although most longitudinal work examining the psychosocial impact of the pandemic do not extend beyond a 4 month follow-up,^44^ our findings are in line with a recent large, longitudinal study of U.S. adults that demonstrated persistent distress and anxiety across the first year of the pandemic.^45^ More long-term longitudinal data is needed to understand within-person changes in COVID-19 exposures, impact, and distress among caregivers and AYA survivors and how it is impacted by pandemic phases including state-level restrictions and outbreaks.^46^ While our findings support differing COVID-19 impacts by race and ethnicity,^22^ we recognize this analysis does not account for the diversity of BIPOC-identified groups. The heterogeneity among BIPOC populations needs to be further explored as well as intersectionality (interactions among race, ethnicity, gender, income, etc.) as related to the psychosocial impact of COVID-19. Furthermore, without a control group, we are unable to determine whether caregivers and AYA survivors experience more negative psychosocial impact compared with their healthy peers or siblings or those with other chronic health conditions. Results from the CEFIS validation samples across various health conditions^31,32^ suggests AYA with cancer and their caregivers may have the lowest COVID-19 psychosocial impact perhaps because they were more prepared for COVID-19 due to their prior medical history and experiences.

Despite these limitations, the CEFIS is a valuable tool for oncology clinicians to understand which patients and families may need greater psychosocial support as the COVID-19 pandemic continues to disrupt various areas of daily life and has lingering impacts on well-being and development. As many families have likely faced prolonged distress, continued support including trauma-informed care may be required beyond the pandemic and can inform our expectations and guide our response to future public health threats. The significant relationships between COVID-19 exposures, impact, and distress support a trauma-informed framework suggesting that those who have been exposed to more COVID-19-related events are more vulnerable to poor psychosocial outcomes. Findings also suggest that difficulty accessing essentials (e.g., food, medicine, healthcare) was the most significant stressor and is important to assess in clinical care. Furthermore, emotional (anxiety, mood, loneliness) and physical (sleep, exercise, diet, sedentary behavior) well-being showed the strongest associations with distress and may represent important targets for behavioral and psychosocial intervention. These findings can inform assessment and intervention for the range of COVID-19-related stressors experienced by caregivers and AYA survivors.

## Supplementary Material

supplementary doc

Additional supporting information can be found online in the Supporting Information section at the end of this article.

## Figures and Tables

**TABLE 1:** Sample characteristics.

	Caregivers (*n* = 422) Mean (SD), range	AYA (*n* = 531) Mean (SD), range
Age (years)	48.55 (7.51), 21–74	19.41 (2.60), 15–28

Child age (years)	15.95 (5.46), 1–25	–
	*n* (valid %)	*n* (valid %)

Race		
American Indian or Alaskan Indian	1 (0.2)	2 (0.4)
Asian	14 (3.3)	20 (3.8)
Black or African American	32 (7.6)	46 (8.7)
Other/More than One Race	35 (9.6)	85 (16.0)
White	283 (77.5)	377 (71.1)

Ethnicity		
Non-Hispanic	326 (89.3)	430 (81.0)
Hispanic	39 (10.7)	101 (19.0)

Gender		
Female	354 (83.9)	276 (52.1)
Male	67 (15.9)	249 (47.0)
Other gender identity	1 (0.2)	5 (0.9)

Child gender		–
Female	206 (48.8)	
Male	213 (50.5)	
Other gender identity	3 (0.7)	

Household income		–
<$24,999	25 (8.4)	
$25,000–$49,999	35 (11.8)	
$50,000–99,999	82 (27.7)	
>$100,000	154 (52.0)	

Study site		
CHOP	349 (82.7)	305 (57.4)
CCHMC	38 (9.0)	135 (25.4)
CHLA	35 (8.3)	91 (17.1)

CEFIS version		
Spanish	10 (2.4)	0 (0.0)
English	412 (97.6)	531(100.0)

Cancer diagnosis		
Leukemia/lymphoma	220 (52.1)	285 (53.7)
Solid tumor	156 (37.0)	189 (35.6)
CNS tumor	46 (10.9)	57 (10.7)

Treatment status		
On active treatment	90 (21.3)	35 (6.6)
Recently off treatment (<2 years)	58 (13.7)	21 (4.0)
Long-term survivorship (≥2 year)	274 (64.9)	475 (89.5)

Abbreviations: CCHMC, Cincinnati Children’s Hospital Medical Center; CHLA, Children’s Hospital Los Angeles; CHOP, Children’s Hospital of Philadelphia; CNS, Central Nervous System; SD, standard deviation.

**TABLE 2:** COVID outcomes by gender.

Caregiver	Female (*n* = 354)	Male (*n* = 67)	*t*	*p*	*d*
COVID-related exposures	7.55 (3.12)	6.90 (2.43)	1.91	.059	0.22
**COVID mean impact**	2.58 (0.62)	2.28 (0.63)	3.15	**.002**	0.49
Caregiver distress	5.26 (2.35)	4.83 (2.17)	1.37	.171	0.18
**Child distress**	5.28 (2.45)	4.56 (2.46)	2.19	**.029**	0.29
AYA	Female (*n* = 274)	Male (*n* = 248)	*t*	*p*	*d*
**COVID-related exposures**	8.49 (3.25)	7.00 (3.06)	5.42	<**.001**	0.47
**COVID mean impact**	2.70 (0.58)	2.53 (0.57)	2.98	**.003**	0.30
**Distress**	5.41 (2.36)	4.30 (2.29)	5.43	<**.001**	0.48

Results presented as *M*(SD). Bold values represent significant differences between groups *p* < .05. AYA, adolescent/young adult

**TABLE 3:** COVID outcomes by race and ethnicity.

Caregiver	NHW (*n* = 269)	BIPOC (*n* = 94)	*t*	*p*	*d*
COVID-related exposures	7.35 (2.77)	8.05 (3.75)	1.68	.096	0.23
**COVID mean impact**	2.62 (0.56)	2.40 (0.77)	2.25	**.027**	0.35
**Caregiver distress**	5.01 (2.20)	5.77 (2.25)	2.77	**.006**	0.33
Child(ren) distress	5.18 (2.31)	5.52 (2.84)	1.02	.308	0.13
AYA	NHW (*n* = 347)	BIPOC (*n* = 181)	*t*	*p*	*d*
COVID-related exposures	7.68 (3.04)	7.99 (3.64)	0.98	.330	0.09
COVID mean impact	2.62 (0.60)	2.63 (0.57)	0.17	.863	0.02
**Distress**	4.73 (2.29)	5.22 (2.54)	2.24	**.025**	0.21

Results presented as *M*(SD). Bold values represent significant differences between groups *p* < .05.

Abbreviations: AYA, adolescent/young adult; BIPOC, Black, Indigenous and People of Color, including Hispanic; NHW, non-Hispanic white.

**TABLE 4:** CEFIS and CEFIS-AYA: Part 3 qualitative categories, frequencies, and notable quotes.

Category	Caregiver (*n* = 322) n (%)	Caregiver quotes	AYA (*n* = 433) n (%)	AYA quotes
Developmental			17 (3.9%)	“Since COVID hit my parents treated me like a fragile child rather than an adult with his own life to live.” (AYA LT Survivor, age 19 years)
Education	152 (47.2%)	“With my son’s chronic medical condition, he was able to do virtual school without feeling like he missed out on something at school.’” (CG of AYA LT Survivor, age 21 years)	117 (27.0%)	“Online schooling was a hard transition and brought upon a huge lack of motivation.” (AYA LT Survivor, age 21 years)
Family	150 (46.6%)	“My wife and I felt like we were given an opportunity to have more quality time with our children. It was stressful; but the forced isolation brought our family back to the dinner table and gave us time that we otherwise wouldn’t have had. So, in some ways, it made our family stronger.” (CG of AYA LT Survivor, age 16 years)	186 (44.1%)	“COVID caused my family to be together a lot more than usual which was a good and bad thing. We were all trying to work, study, and do daily tasks in our house at the same time. It could get noisy, making it hard to concentrate. We enjoyed doing more things like playing board games as a family at home that we usually wouldn’t get to do.“ (AYA On-Treatment, age 22 years)
Lifestyle	139 (43.2%)	“We are part of a church community that has not been able to meet. Additionally, there has been tension related to racial issues escalated by the racial tensions in our society that haven’t been handled in healthy ways, partially due to the pandemic and our inability to be physically together. This has been very painful for us as a family.” (CG of Child Recently Off Treatment, age 3 years)	138 (31.9%)	“I had to move home from college so that I could be safe considering my cancer history.” (AYA LT Survivor, age 22 years)
Emotional well-being	133 (41.3%)	“Both of my kids depression and anxiety increased during COVID-19. They both experienced challenges with sleeping and getting rest. Both suffered from lack of personal relationships and the ability to go places and do things. The stress level for my husband who is a teacher is sky high.” (CG of AYA LT Survivor, age 19 years)	96 (22.2%)	“The biggest effect is the continuous anxiety that comes as a result of being someone with a compromised immune system and the risk that I could potentially be exposed.” (AYA LT Survivor, age 25 years)
Social well-being	132 (41.0%)	“Our children were more upset about being restricted from seeing friends… so the isolation was the toughest part for them.” (CG of Child LT Survivor, age 11 years)	110 (25.4%)	“I have been able to establish means of virtual communication with friends, which will be effective later when we move away from each other.” (AYA LT Survivor, age 18 years)
Work	79 (24.5%)	“I typically travel A LOT for work; being grounded and having my kids at home has let us spend a lot more time together. Caring for my daughter with her bone tumor diagnosis would have been very disruptive to my job if it hadn’t been for COVID.” (CG of AYA On-Treatment, age 18 years)	103 (23.8%)	“The search for a job post-graduation has been difficult and not the way I expected it to go. I am stuck between giving myself time to take off and focus on myself and taking advantage of this time by trying to find something. This isn’t the way I wanted to start working.” (AYA LT Survivor, age 22 years)
Health behaviors	66 (20.5%)	“My son (the patient) continued with his college co-op throughout [the pandemic], working in person daily. His 2 roommates moved back home, and he had the house to himself, so he exercised more and ate better with meal planning.” (CG of AYA LT Survivor, age 22 years)	102 (23.6%)	“A positive is that I have been able to get into a better exercise routine. Without needing to go places every day, I can exercise more effectively.” (AYA LT Survivor, age 18 years)
Physical health	64 (19.9%)	“I think as a mother of a child with cancer during a pandemic, I am extra, extra cautious and extra nervous, which puts more stress on my child who wants to feel and live normal.” (CG of AYA On-Treatment, age 14 years)	96 (22.2%)	“Overall, the experience of COVID-19 has a lot of parallels to when I re-introduced myself back into the world after treatment (e.g., wearing a mask, not going to school)… except this time everyone is doing it with me. So, I’ve felt somewhat prepared for COVID even though everyone else wasn’t.” (AYA LT Survivor, age 16 years)

Abbreviations: AYA, Adolescent/Young Adult; CG, Caregiver; LT Survivor, long-term survivor.

## Data Availability

Data is available upon request from the corresponding author.

## Abbreviations:

AYA: adolescent and young adult
BIPOC: Black, Indigenous and People of Color
CEFIS: COVID-19 Exposure and Family Impact Survey
CNS: central nervous system
NHW: non-Hispanic white
