# Supplementary material for: Psychosocial impact of COVID-19 on caregivers and adolescents and young adult survivors of childhood cancer
Source: Pediatr Blood Cancer. Author manuscript; Available in PMC 2026 Feb 26. (PMC12944686; doi:10.1002/pbc.30291)
Supplement: supplementary doc [file NIHMS2085561-supplement-supplementary_doc.docx]

**TABLE S1: Description of studies and recruitment methods**

| **Study Name** | **Study Purpose** | **Patient Eligibility** | **Caregiver Eligibility** | **Recruitment Methods** | **Study Design/Requirements** | **Language** | **Sample Included in analysis** |
| --- | --- | --- | --- | --- | --- | --- | --- |
| **ADAPTS**  **Adherence Assessments and Personalized Timely Support** | Determine time-varying contextual factors (e.g., symptoms, affect) that influence oral chemotherapy adherence among AYA | -AYA ages 14-25, with acute lymphoblastic leukemia and lymphomas in maintenance taking 6-MP  -at least 6 months of maintenance treatment remaining  -absence of any cognitive impairment | -Caregiver of a study participant who lives in the same household with their AYA at least 50% of the time | -Primarily in clinic recruitment by study coordinator with the option of remote recruitment by phone | -6-month study, with bursts of daily surveys and daily use of a MEMS TrackCAP  -Baseline questionnaire via REDCap  -monthly check-in with participants before or after an already scheduled clinic appointment | English | CEFIS-AYA (n=17)  CEFIS (n=19) |
| **AYA Mentorship Program**  **A Pilot Investigation of a Peer-to-Peer Mentoring Program for Adolescent and Young Adults with Cancer** | Pairing AYA with a YA cancer survivor mentor | Mentees  -14-29 years old,  -Receiving cancer treatment or within one year of completing cancer treatment  Mentors  -18 years old or older  -Completed cancer treatment one year ago or more  -Nominated by member of their oncology team | N/A | -Referrals from clinicians (oncologists or psychosocial)  -Recruited by study coordinator in clinic or over the phone if they didn’t have an upcoming appointment | -Check ins 2x/month for 3 months, and then follow up on their own schedule for 9 months  -3 questionnaires completed at baseline, 3 months and 12 months | English | CEFIS-AYA (n=10)  CEFIS (n=0) |
| **AYA TEAMS**  **AYA Tracking Engagement and Management Skills**  **(Multisite study including Children’s Hospital Los Angeles (CHLA) and Cincinnati Children’s Hospital Medical Center (CCHMC))** | Examine the predictors and patterns of transition readiness, self-management skills and engagement in long-term follow-up over time in AYA survivors of childhood cancer | -AYA 16-25 years  -At least 5 years from diagnosis and 2 years from end of treatment  -Visited CHOP, CHLA, or CCHMC within the previous 18 months;  -Cognitively capable to complete measures, as determined by the medical team and/or medical record review | -Parent or caregiver of enrolled AYA participant  -caregivers of married AYA are not eligible | -Study team identified potentially eligible patients via clinic lists  -Recruited in person by study coordinator in survivorship clinic or remotely via phone | -AYA participants complete three annual online surveys via REDCap  -If AYA are transferred to adult care they complete two surveys related to their transition | AYA – English Only  Caregivers - English or Spanish | CEFIS-AYA (n=471)  CEFIS English (n=252)  CEFIS Spanish (n=10) |
| **CARES**  **Check-In About Recent Experiences and Strengths** | Clinical program to assess psychosocial needs at end of treatment and provide educational materials to families | -Confirmed cancer diagnosis  -Completed cancer treatment that included radiation and/or chemotherapy within the last 1 year  -Patients completed surveys if >age 11 | -Caregivers of patients were eligible to participate whether their child was over or under 18 | -Caregivers or adult patients (ages 18 or older) were notified of program prior to a scheduled appointment and approached at appointment | -review end of treatment educational handbook and completed screening questionnaires | English | CEFIS-AYA (n=19)  CEFIS (n=56) |
| **HCST Sleep**  **Sleep in Hematopoietic Stem Cell Transplant Patients** | Test the feasibility of extended time between vitals checks for patients undergoing hematopoietic stem cell transplant (HCST) | -Ages 8-21 undergoing HSCT at CHOP  -absence of cognitive impairment/developmental disability that impacts the ability to self-report  -absence of previously diagnosed sleep disorder | -Caregiver and legal guardian of a study participant | -Families were introduced to the study by clinic staff and if interested contacted and consented by a research assistant  -Patients were recruited in-patient in person | -Study lasted for 16 days—with daily remote measures/wear an actigraph, and then visits every 5 days (in person or remote)  -Caregivers completed surveys via REDCap  -No CEFIS-AYA report | English | CEFIS-AYA (n=0)  CEFIS (n=18) |
| **I-CAN**  **Adolescent and Young Adult Involvement in Cancer Treatment Responsibilities** | Understand how AYAs and caregivers determine and allocate cancer-related responsibilities | -Ages 9-25 with confirmed cancer diagnosis  -receiving active treatment (defined as chemo, radiation, or both)  -at least 1 month post cancer diagnosis | -Caregiver of a study participant who lives in the same household with their AYA at least 50% of the time | -Patients were approached in clinic and remotely by study staff | -One time interview (~60 min in person or remote) with AYA-caregiver dyads  -Questionnaire completed via REDCap | English | CEFIS-AYA (n=10)  CEFIS (n=21) |
| **TIPS**  **Training in Problem Solving for maternal caregivers of young adult survivors of a childhood brain tumor** | To evaluate the preliminary efficacy, acceptability, and feasibility of an adapted problem-solving intervention for caregivers of young adult survivors of a childhood brain tumor | -Waiver of consent to review young adult survivor (YAS) medical record  -Ages 18-29  -non-genetically based childhood brain tumors  -No developmental delays  -2-years post-completion of treatment and living at home | -Maternal caregivers of young adult survivor meeting patient eligibility criteria  -Condition Management Ability screening score of <51 | -Study team identified potentially eligible patients via clinic lists and CHOP Tumor registry reports  -Recruitment primarily took place via phone by study coordinator | -Randomized-controlled trial with 2 Intervention Arms: Enhanced Usual Care (Access to paper/electronic resources with 2 interviews) and Training in Problem-Solving (5 sessions, 5-check-in sessions with Coach)  -Surveys administered at baseline, mid- and post-intervention  -CEFIS administered post-intervention only | English | CEFIS AYA (n=0)  CEFIS (n=6) |
| **TrACC**  **Tracking Adjustment, Communication, and Change - Impact of Pediatric Germline Testing in a Pediatric Cancer Predisposition** | Examine the psychosocial impact of genetic testing for cancer predisposition among youth and families from pre- to post-testing | -Patients age 25 and under undergoing genetic cancer predisposition testing at CHOP  -Without a terminal diagnosis  -Patients age 12-25 complete measures | -First and/or Second caregivers of an eligible patient | -Patients were introduced to the study via genetic counselors at the first visit to the CHOP Cancer Predisposition Clinic  -Study staff approached in clinic or remotely by phone for consent | -Participation lasted approximately 8 months  -Three surveys (1 pre and 2 post genetic testing)  -1 qualitative interview if the child proband tested positive for a genetic cancer predisposition | English | CEFIS-AYA (n=4)  CEFIS (n=40)  *NOTE CEFIS and CEFIS-AYA responses were excluded if the patient did not have a current/prior confirmed cancer diagnosis |

**TABLE S2 COVID Outcomes by Cancer Type**

| **Caregiver** | **Leukemia/**  **Lymphoma**  **(n=220)** | **Solid Tumor (n=156)** | **CNS Tumor**  **(n=46)** | ***F*** | ***p*** | ***η^2^*** |
| --- | --- | --- | --- | --- | --- | --- |
| COVID-Related Exposure | 7.24 (3.01) | 7.46 (3.05) | 8.35 (2.88) | 2.60 | .076 | .012 |
| **COVID Mean Impact** | **2.43 (0.63)** | **2.58 (0.65)** | **2.88 (0.42)** | **7.88** | **<.001 ^a^** | **.050** |
| Caregiver Distress | 5.17 (2.32) | 5.14 (2.36) | 5.50 (2.20) | 0.46 | .630 | .002 |
| Child(ren) Distress | 5.08 (2.38) | 5.08 (2.62) | 5.96 (2.22) | 2.61 | .075 | .012 |
| **AYA** | **Leukemia/**  **Lymphoma**  **(n=285)** | **Solid Tumor**  **(n=189)** | **CNS Tumor**  **(n=57)** | ***F*** | ***p*** | ***η^2^*** |
| COVID-Related Exposure | 7.81 (3.31) | 7.73 (3.15) | 7.86 (3.43) | 0.05 | .949 | <.001 |
| COVID Mean Impact | 2.64 (0.58) | 2.64 (0.55) | 2.53 (0.72) | 0.62 | .537 | .003 |
| Distress | 4.83 (2.36) | 4.95 (2.40) | 5.05 (2.53) | 0.28 | .754 | .001 |

Results presented as *M(SD)*

Bold values represent significant main effects

^a^CNS Tumor group significantly different from Leukemia/Lymphoma and Solid Tumor group (*p*< 0.05)

Abbreviations: CNS=Central Nervous System

**TABLE S3 COVID Outcomes by Treatment Status**

| **Caregiver** | **Currently On-Treatment**  (n=90) | **Recently Off-Treatment**  (n=58) | **Long-term Survivorship**  (n=274) | ***F*** | ***p*** | *η^2^* |
| --- | --- | --- | --- | --- | --- | --- |
| COVID-Related Exposures | 7.54 (2.99) | 6.88(2.72) | 7.53(3.09) | 1.16 | .314 | .006 |
| COVID Mean Impact | 2.65 (0.61) | 2.40 (0.61) | 2.52 (0.64) | 2.37 | .095 | .016 |
| Caregiver Distress | 5.57 (2.24) | 5.10 (2.36) | 5.09 (2.33) | 1.50 | .225 | .007 |
| Child(ren) Distress | 5.33 (2.62) | 4.64(2.50) | 5.24 (2.40) | 1.65 | .193 | .008 |
| **AYA** | **Currently On-Treatment**  (n=35) | **Recently Off-Treatment**  (n=21) | **Long-term Survivorship**  (n=475) | ***F*** | *p* | ***η^2^*** |
| COVID-Related Exposures | 7.03 (3.05) | 6.62 (3.43) | 7.90 (3.25) | 2.58 | .076 | .010 |
| COVID Mean Impact | 2.45 (0.42) | 2.51 (0.64) | 2.65 (0.59) | 1.53 | .219 | .008 |
| **Distress** | **4.11 (2.62)** | **3.15 (2.11)** | **5.03 (2.34)** | **8.14** | **<.001 ^a^** | **.030** |

Results presented as M(SD)

Bold values represent significant main effects

^a^Long-term Survivorship group significantly different than recently off-treatment group (*p*< 0.05)

Abbreviations: CNS=Central Nervous System

| **TABLE S4** CEFIS and CEFIS-AYA: Qualitative Category and Sub-Category Definitions, Frequencies and Additional Quotes | | | | | |
| --- | --- | --- | --- | --- | --- |
| **CATEGORY** | **DEFINITION.** *Responses related to …* | **CG** n (%) | **AYA** n (%) | **CAREGIVER QUOTES** | **AYA QUOTES** |
| **DEVELOPMENTAL** | **Adolescence to emerging adulthood.** |  | **17 (3.9%)** |  | “It was nice to be back home, but it also took away a lot of my freedom and independence that I had grown accustomed to.” (AYA LT Survivor, Age 21) |
| Independence | Dependence/autonomy from family of origin |  | 13 (3.0%) |  |  |
| **EDUCATION** | **Childcare, pre-k, k-12, or higher education** | **152 (47.2%)** | **117 (27.0%)** | “All three kids are doing virtual schooling. While two of them have handled it very well, one has had a really hard time. His grades have slipped. I don’t feel like they’re learning as much as they would have if COVID-19 hadn’t happened. (CG of AYA LT Survivor, Age 16) | “I don’t mind online school. I’m online for my main school and hybrid for tech school. I feel a little more focused and organized online. Even though I feel lonelier, I see friends twice a week at tech school and we talk via phone. It’s a lot easier and I love it. (AYA LT Survivor, Age 16) |
| School Arrangements | Modes of education (in-person, virtual, or hybrid) | 107 (33.2%) | 75 (17.3%) |  |  |
| Learning | Acquiring academic knowledge and skills. | 36 (11.2%) | 33 (7.6%) |  |  |
| **FAMILY** | **Immediate and Extended Family** | **150 (46.6%)** | **186 (44.1%)** | “We actually felt like we had a bit of a gift in that we were able to spend more time together as a family and felt more present during conversations.” (CG of AYA LT Survivor, Age 19) | “It brought our family closer since I returned home from college. I was able to bond and connect more with my family and got closer to my siblings.” (AYA Survivor, Age 19); “COVID has made my family not get along well because seeing each other every day got annoying and nothing new ever happened.” (AYA On-Treatment, Age 18) |
| Time with Family | Quantity of time spent with family | 95 (29.5%) | 103 (23.8%) |  |  |
| Relationships with Family | Quality of family relationships | 36 (11.2%) | 81 (18.7%) |  |  |
| Family/Household Management | Ability to manage family routines and household tasks/chores | 19 (5.9%) |  |  |  |
| **LIFESTYLE** | **Everyday activities** | **139 (43.2%)** | **138 (31.9%)** | "Prior to COVID, we went to work, came home tired, stressed and irritable only to do chores and prepare for the next day outside the home. COVID, although scary at times, helped me to imagine how I would prefer to live my life." (CG of AYA LT Survivor, Age 17) | "I feel like it has derailed my life. I am set to be a senior [in high school] in a few months and everything that I've been looking forward to is canceled. I miss normal life." (AYA LT Survivor, Age 17) |
| Regular/Leisure Activities | General leisure activities (e.g., extracurriculars, daily routines) | 79 (24.5%) | 72 (16.6%) |  |  |
| Milestones/Major Life Events | Occurrences that mark a significant life event (e.g., graduation) | 31 (9.6%) | 24 (5.5%) |  |  |
| Living Arrangements | Changes to people living with and/or location | 29 (9.0%) | 38 (8.8%) |  |  |
| **EMOTIONAL WELL-BEING** | **Psychological Health** | **133 (41.3%)** | **96 (22.2%)** | "My son was diagnosed with cancer during the pandemic, so as if the pandemic wasn't stressful enough, then having a child diagnosed with cancer on top of it caused a great deal of stress on our immediate as well as our extended family." (CG of Child On-Treatment, Age 9) | We were all feeling the anxiety of not knowing what the future could be and not knowing when the world would go back to normal ... overall, we're doing fine emotionally, but it is definitely something that if we think about what could have been this year then we tend to get upset/depressed for a time." (AYA LT Survivor, Age 18) |
| General Distress | Non-specific general reports of mental strain and stress | 53 (16.5%) | 50 (11.5%) |  |  |
| Anxiety and Worry | Temporary or sustained feelings of uneasiness, nervousness, fear | 48 (14.9%) | 23 (5.3%) |  |  |
| Mood | Low mood, sadness, and depression | 23 (7.1%) | 9 (2.1%) |  |  |
| **SOCIAL WELL-BEING** | **Friends and peer groups** | **132 (41.0%)** | **110 (25.4%)** | “Our main hardship has been one of isolation. Both children have not seen friends for over a year and did not compete on their dance team while other children did. They miss socializing.” (CG of AYA LT Survivor, Age 16) | “My biggest problem with COVID is loneliness.” (Female Survivor, Age 16); “I had to move home, and this wasn’t a good time for me because I am Queer and not out to my family yet, so I had to hide part of myself for months when I had already been living my life as my true self at [college].” (AYA LT Survivor, Age 20) |
| Time with Friends | Quantity of time spent with friends and peers | 51 (15.8%) | 47 (10.9%) |  |  |
| Isolation and Loneliness | Being alone and feeling separated from peers | 48 (14.9%) | 30 (6.9%) |  |  |
| Relationships with Friends | Quality of friendships | 3 (0.9%) | 15 (3.5%) |  |  |
| Romantic Relationships and Dating | Relationships with partners or meeting new partners |  | 9 (2.1%) |  |  |
| **WORK** | **Profession, occupation, trade, or vocation** | **79 (24.5%)** | **103 (23.8%)** | I am an essential employee and am working outside of the home. It makes it difficult to help the kids with their [virtual] schoolwork while I'm at work." (CG of AYA LT Survivor, Age 16) | "I am immunocompromised, so I had to leave my job to keep myself at lower risk." (AYA LT Survivor, Age 24) |
| Work Arrangements | Changes to working patterns and/or locations (remote/in-person) | 44 (12.7%) | 32 (7.4%) |  |  |
| Employment and Income | Changes to employment status and income | 35 (10.9%) | 63 (14.5%) |  |  |
| **HEALTH BEHAVIORS** | **Actions that affect health** | **66 (20.5%)** | **102 (23.6%)** | "Our child who had cancer last year was able to sleep a lot since school was closed, which helped him shake the lingering chemo fatigue." (CG of AYA Recently Off-Treatment, Age 18  “We had more time to cook and eat healthy.” (CG of AYA LT Survivor, Age 21) | “I ate, slept, and consumed substances much more than before COVID.” (Female Survivor, Age 22); “Quarantine and the state shutdown made me more anxious about COVID and its parallels to my cancer treatment.” (AYA LT Survivor, Age 17) |
| COVID-Related Behaviors | Behavioral response to COVID (mask wearing, social distancing) | 35 (10.9%) | 48 (11.1%) |  |  |
| Physical Activity | Movement behaviors/exercise routines | 11 (3.4%) | 23 (5.3%) |  |  |
| Nutrition | Diet/eating behaviors | 6 (1.86%) | 10 (2.3%) |  |  |
| Sedentary Behavior | Low energy expenditure activities (e.g., sitting/ screen time) | 7 (2.17%) | 8 (1.8%) |  |  |
| Sleep | Amount and quality of sleep; falling/staying asleep; napping | 7 (2.17%) | 8 (1.8%) |  |  |
| Substance Use | Abstinence from, dependence on, or abuse of substances. |  | 2 (0.5%) |  |  |
| **PHYSICAL HEALTH** | **Physical health status and risk** | **64 (19.9%)** | **96 (22.2%)** | "COVID interrupted my children's access to healthcare needs. We were unable to access dental and medical care especially in the first 6-8 months of COVID-19. It has put a lot of stress and anxiety on the family." (CG of Child On-Treatment, Age 10) | "COVID-19 has made me scared about how, if I ever caught it, my immune system would be impacted as I'm not sure what my cancer diagnosis has done to my body and what my chances would be with COVID-19." (AYA LT Survivor, Age 17) |
| COVID Risk Perception/Uncertainty | Perceived risk of infection or uncertainty about severity of illness | 33 (10.2%) | 35 (8.1%) |  |  |
| Cancer Care | Cancer-specific health care | 12 (3.7%) | 6 (1.4%) |  |  |
| COVID Exposure/Symptoms/Diagnosis | Exposure, diagnosis, symptoms, or death from COVID. | 12 (3.7%) | 41 (9.5%) |  |  |
| Non-COVID Illness/Ailments | Diagnosis, treatment, or death from non-COVID health concern | 5 (1.6%) | 6 (1.4%) |  |  |

Abbreviations: AYA=Adolescent/Young Adult; CG=Caregiver; LT Survivor=Long-term survivor

**FIGURE S1** Caregiver (A) and AYA (B) COVID-19 Direct Virus Exposures

Abbreviations: ICU=intensive care unit.

Note: Other Relatives=extended family members; Multiple Family Members=more than one

family member not otherwise specified (e.g. “all of us”).

| **FIGURE S2** Caregiver (A) and AYA (B) CEFIS Outcomes Across Time    CG=caregiver  Note: Exposure range for caregivers 0-25, exposure range for AYA 0-28; Impact range: 1-4; Distress range: 1-10 |
| --- |
